# A Simplified Kinetic Modeling of CO_2_ Absorption into Water and Monoethanolamine Solution in Hollow-Fiber Membrane Contactors

**DOI:** 10.3390/membranes13050494

**Published:** 2023-05-05

**Authors:** Mai Lien Tran, Chi Hieu Nguyen, Kuan-Yan Chu, Ruey-Shin Juang

**Affiliations:** 1 Institute of Environmental Science, Engineering and Management, Industrial University of Ho Chi Minh City, Ho Chi Minh City 700000, Vietnam; tranmailien@iuh.edu.vn (M.L.T.); nguyenchihieu_mt@iuh.edu.vn (C.H.N.); 2 Department of Chemical and Materials Engineering, Chang Gung University, Guishan, Taoyuan 33302, Taiwan; s955244@mail.yzu.edu.tw; 3 Department of Internal Medicine, Division of Nephrology, Chang Gung Memorial Hospital Linkou, Taoyuan 33305, Taiwan; 4 Department of Safety, Health and Environmental Engineering, Ming Chi University of Technology, Taishan, New Taipei City 24301, Taiwan

**Keywords:** kinetic modeling, CO_2_ absorption, monoethanolamine, hollow-fiber membrane contactors

## Abstract

The absorption of CO_2_ from CO_2_-N_2_ gas mixtures using water and monoethanolamine (MEA) solution in polypropylene (PP) hollow-fiber membrane contactors was experimentally and theoretically examined. Gas was flowed through the lumen of the module, whereas the absorbent liquid was passed counter-currently across the shell. Experiments were carried out under various gas- and liquid-phase velocities as well as MEA concentrations. The effect of pressure difference between the gas and liquid phases on the flux of CO_2_ absorption in the range of 15–85 kPa was also investigated. A simplified mass balance model that considers non-wetting mode as well as adopts the overall mass-transfer coefficient evaluated from absorption experiments was proposed to follow the present physical and chemical absorption processes. This simplified model allowed us to predict the effective length of the fiber for CO_2_ absorption, which is crucial in selecting and designing membrane contactors for this purpose. Finally, the significance of membrane wetting could be highlighted by this model while using high concentrations of MEA in the chemical absorption process.

## 1. Introduction

Carbon dioxide has been proven to be the largest contribution of greenhouse gases, which results in the increase in the earth’s surface temperature. It is also reported that half of CO_2_ emissions are produced by power plants using fossil fuels [1,2,3]. Hence, the development of an efficient separation process is highly desired to remove CO_2_ from the places where CO_2_ is generated. In general, the bubble column, packed tower, venturi scrubber, and sieve tray column can be used for this purpose. The commercial process that is widely used for CO_2_ separation is the packed column, but new technology is still required because the packed towers, for example, have many advantages such as channeling, flooding, and large-scale equipment. The gas absorption process using membrane units is considered as an alternative to recover CO_2_ from waste gas streams. The hollow-fiber membrane contactor (HFMC) offers a much larger contact area per unit volume in comparison with packed and tray columns and has the advantages of no entrainment, no foaming, and no restrictions on operating flow rates [2,3,4,5,6,7,8,9].

Alkanolamines including monoethanolamine (MEA), dithenaolamine (DEA), *N*-methyl-diethanolamine (MDEA), di-2-propanolamine, and 2-amino-2-methyl-l-propanol (AMP) are dominantly used as absorbent liquids for CO_2_ removal due to their high reaction rates [10,11,12,13,14]. Using HFMCs, Yeon et al. [15] have examined the absorption of CO_2_ in PVDF and PTFE modules using single MEA absorbent. Wang et al. [16] have theoretically studied the capture of CO_2_ by three solutions of MDEA, AMP, and DEA in HFMCs with a non-wetting mode. In addition, Yeon et al. [17] have examined the rate of CO_2_ absorption in PP module using the mixed absorbent liquids of piperazine (PZ) and triethanolamine (TEA).

Various kinetic models have been developed in the literature to follow the absorption of CO_2_ in HFMCs by absorbent liquids [8,9,15,16,18,19,20,21,22,23,24,25,26,27]. Most of them have used the complicated numerical methods to solve a set of differential mass balance equations to predict the change of CO_2_ concentration along the hollow fibers. For example, Kim and Yang [18] have adopted a set of governing equations with common assumptions that the velocity in the lumen side can be described as fully developed parabolic profile and that the velocity through the shell side can be characterized by the free surface model of Happel [26]. Yeon et al. [15] have also employed differential mass balance equations to describe diffusion and forced convection in a medium that flows laminarly in the lumen side. They have assumed an irreversible reaction of CO_2_ taking place with MEA and determined the mass transfer rates of CO_2_ in PVDF and PTFE hollow fibers. Generally speaking, the mathematical complexity of numerical methods used for solving a set of differential mass balance equations makes the kinetic model somewhat practically inconvenient.

The absorption of CO_2_ from a synthetic 15 vol% CO_2_-N_2_ gas mixture by using water and MEA solutions was investigated in HFMCs. The effects of liquid- and gas-phase velocities as well as the pressure difference between liquid and gas phases on the flux of CO_2_ absorption were explored. A simplified model involving mass balance equations-only was proposed to follow the absorption process of CO_2_ and to estimate the effective length of the fiber, in which the non-wetting mode was assumed and the overall mass-transfer coefficient based on physical and chemical absorption was considered.

## 2. Kinetic Modeling

### 2.1. Determination of Overall Mass-Transfer Coefficient

The mass transfer between gas and liquid phases through HFMC occurs in three parts: stagnant gas film, the membrane itself, and stagnant liquid film [15]. The overall rate of CO_2_ absorption, *R*_A_ (mol s^−1^), is expressed by Equation (1):

(1) $$R_{A}=K_{L}A_{T}\Delta C_{\mathrm{lm}}=Q_{g}\left(C_{g,\mathrm{out}}-C_{g,\mathrm{in}}\right)$$

where *K*_L_ is the overall mass-transfer coefficient based on liquid phase (m s^−1^); *A*_T_ is the effective contact area (m^2^); *Q*_L_ is the volumetric flow rate of liquid phase (m^3^ s^−1^); and *C*_g,out_ and *C*_g,in_ are the gas-phase concentrations of CO_2_ in the outlet and inlet of the module, respectively (mol m^−3^). Additionally, Δ*C*_lm_ is the logarithmic mean concentration difference, which is expressed by the following equation:

(2) $$\Delta C_{\mathrm{lm}}=\frac{\left(HC_{g,\mathrm{in}}-C_{L,\mathrm{out}}\right)-\left(HC_{g,\mathrm{out}}-C_{L,\mathrm{in}}\right)}{ln\left[\left(HC_{g,\mathrm{in}}-C_{L,\mathrm{out}}\right)/\left(HC_{g,\mathrm{out}}-C_{L,\mathrm{in}}\right)\right]}$$

Here, the value of *H* for CO_2_ in water is adopted to be 2.916 kPa m^3^ mol^−1^ [28], whereas it is taken to be 3.564 kPa m^3^ mol^−1^ in 0.005–0.01 M of MEA solution and becomes 3.518 kPa m^3^ mol^−1^ in 1.0 M of MEA solution [29].

### 2.2. Model Development

A simplified mathematical model was developed based on the mass balance concept, combining process conditions, membrane properties, and module geometric characteristics. The following assumptions are made: (a) absorption of single component (CO_2_) from a CO_2_-N_2_ gas mixture flowing through the lumen side of the module into an aqueous solution flowing in the shell side; (b) steady state and isothermal operation; (c) Newtonian fluids with constant physical properties; (d) hydrophobic membrane with non-wetting; and (e) the applicability of Henry’s law [8,9,18,19,21,22,25,26,27].

**Figure 1:**
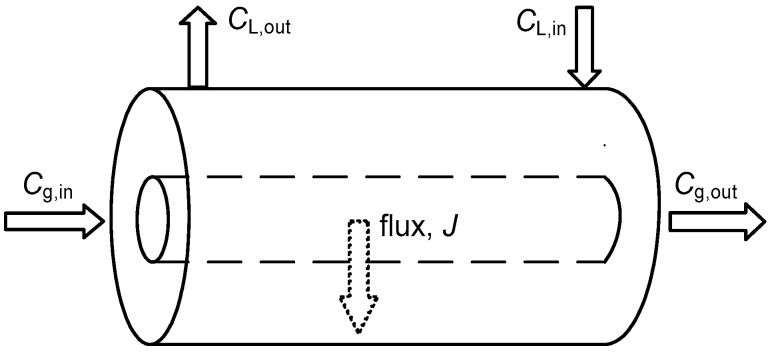
The control volume of gas absorption in hollow fibers.

As shown in Figure 1, the mass balance of CO_2_ within the membrane contactor using water as absorbent liquid is described as follows:

(3) $$Q_{L}C_{L,\mathrm{in}}-Q_{L}C_{L,\mathrm{out}}+J_{A}A_{T}=0$$

where *J*_A_ is the flux of CO_2_ absorption through the hollow fibers (mol m^−2^ s^−1^), which is calculated by the following equation:

(4) $$J_{A}=K_{L}\left(C_{L,\mathrm{in}}-C_{L,\mathrm{out}}\right)$$

where *C*_L_ is the concentration of CO_2_ in liquid phase (mol m^−3^).

Since *J*_A_ depends on the concentration of CO_2_, it must be calculated every time according to Equations (3) and (4) using the MATLAB program (MathWorks, Natick, MA, USA). The number of grids (*N*_z_) in the computational domain of 500 (in the axial direction) was used. Therefore, the value of *C*_L,out_ can be calculated by Equation (5):

(5) $$C_{L,\mathrm{out}}=\frac{Q_{L}C_{L,\mathrm{in}}+K_{L}\left(A_{T}/N_{z}\right)C_{g,\mathrm{in}}}{Q_{L}+K_{L}\left(A_{T}/N_{z}\right)}$$

On the other hand, the mass balance within the membrane contactor using MEA solution as absorbent liquid is described as follows:

(6) $$Q_{L}C_{L,\mathrm{in}}-Q_{L}C_{L,\mathrm{out}}+J_{A}A_{T}-r_{A}V_{\mathrm{shell}}=0$$

where *r*_A_ is the reaction rate between MEA and CO_2_ (mol m^−3^ s^−1^).

In general, carbamate is formed when CO_2_ gas reacts with primary and secondary alkanolamines [13,30,31]:

(7) $$CO_{2}+2R_{1}R_{2}NH⇌R_{1}R_{2}NCO_{2}^{-}+R_{1}R_{2}NH_{2}^{+}$$

where *R*_1_ is an alkyl group, and *R*_2_ is H for primary amines and an alkyl group for secondary amines. The zwitterion mechanism has been commonly used in aqueous alkanolamine solutions [32,33]. The reaction steps successively involve the formation of a zwitterion,

(8) $$R_{1}R_{2}NH+CO_{2}⇌R_{1}R_{2}NH^{+}CO_{2}^{-},\;k_{2,amine}/k_{-1}$$

and the subsequent removal of the proton by a base *B* (base catalysis),

(9) $$R_{1}R_{2}NH^{+}CO_{2}^{-}+B\overset{k_{B}}{\to }R_{1}R_{2}NCO_{2}^{-}+BH^{+}$$

where *B* could be an amine, OH^−^, or H_2_O, although the contribution of OH^−^ can be neglected because its concentration is very low, compared to that of the amine and H_2_O [14].

According to this zwitterion mechanism, the forward reaction rate equation for CO_2_ at quasi-steady state, *r*_A_, has been derived by Danckwerts [34] as follows:

(10) $$r_{A}=\sum \frac{k_{2,\mathrm{amine}}C_{L}C_{\mathrm{amine}}}{1+{\left\{\left[\left(k_{w}/k_{-1}\right)C_{w}\right]+\sum \left[\left(k_{\mathrm{amine}}/k_{-1}\right)C_{\mathrm{amine}}\right]\right\}}^{-1}}$$

So, in the MEA-CO_2_ system we have

(11) $$r_{A}=\sum \frac{k_{2,\mathrm{MEA}}C_{L}C_{\mathrm{MEA}}}{1+{\left\{\left[\left(k_{w}/k_{-1}\right)C_{w}\right]+\left[\left(k_{\mathrm{MEA}}/k_{-1}\right)C_{\mathrm{MEA}}\right]\right\}}^{-1}}$$

where *C*_w_ is equal to 55.5 M. The kinetic parameters of *k*_2,MEA_, (*k*_w_/*k*_−1_), and (*k*_MEA_/*k*_−1_) adopted here at 25 °C are 6.358 m^3^ mol^−1^ s^−1^, 1.507 × 10^−6^ m^3^ mol^−1^, and 2.485 × 10^−4^ m^3^ mol^−1^, respectively [13,25]. In this case, the value of *C*_L,out_ can be calculated by Equation (12):

(12) $$C_{L,\mathrm{out}}=\frac{Q_{L}C_{L,\mathrm{in}}+K_{L}\left(A_{T}/N_{z}\right)C_{g,\mathrm{in}}-r_{A}\left(V_{\mathrm{shell}}/N_{z}\right)}{Q_{L}+K_{L}\left(A_{T}/N_{z}\right)}$$

## 3. Materials and Methods

### 3.1. Materials

MEA was purchased from Aldrich Chemicals Co. A Liqui-Cel microporous hollow-fiber extra-flow 2.5 × 8 module, which was supplied from3M^TM^ Separation and Purification Division. (Charlotte, NC, USA), was used as membrane contactor for CO_2_ absorption in this work. The hollow fibers in this module were X-50 type and made of polypropylene (PP). The characteristics of the membrane contactor are listed in Table 1. Deionized water (Millipore, Milli-Q, Burlington, MA, USA) was used. All chemicals were used without any further purification.

### 3.2. Absorption Experiments

**Figure 2:**
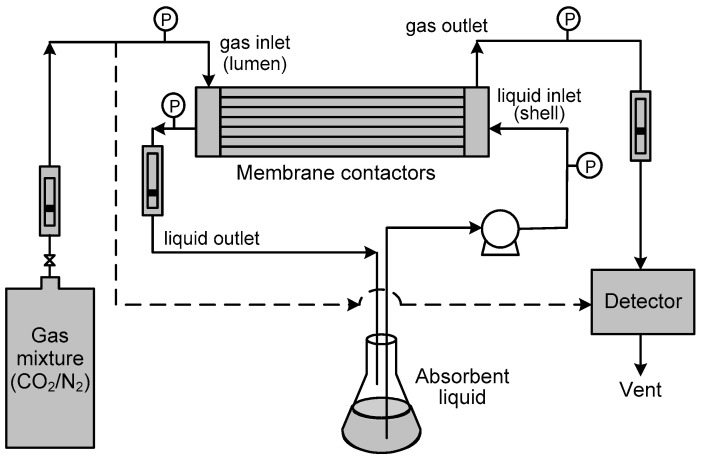
Experimental setup of CO_2_ absorption in Liqui-Cel extra-flow model HFMCs.

The experimental setup for CO_2_ removal is schematically illustrated in Figure 2. The gas containing 15 vol% of CO_2_ (balance N_2_) was passed upstream in the lumen side of the module, and the absorbent liquid was supplied downstream in the shell side. The absorbent liquids used in this study were deionized water and the aqueous solution containing 0.005–1.0 M MEA, which had a volume of 1 L otherwise stated elsewhere. The gas-phase velocity *u*_g_ was varied in the range of 0.041–0.124 m s^−1^, and the liquid-phase velocity *u*_L_ changed in the range of 0.008–0.02 m s^−1^. The pressure difference of the liquid and gas phases was changed in the range of 15–85 kPa by a needle valve to form the stable gas-liquid interface within the module. The gases coming from the absorption were sampled and analyzed by TCD-GC (Shimadzu, GC-14B, Kyoto, Japan) at pre-set time intervals. After each run, the deionized water (2 dm^3^) was poured into both sides of the membrane contactor to remove the absorbent liquids. Then, ethanol (0.5 dm^3^) was flowed through both sides of the module to remove water in the pores of the membrane. Finally, N_2_ gas went through both sides of the module for 30 min.

## 4. Results and Discussion

### 4.1. Effect of Fluid Velocity on K_L_ without Absorbent Recycling

**Figure 3:**
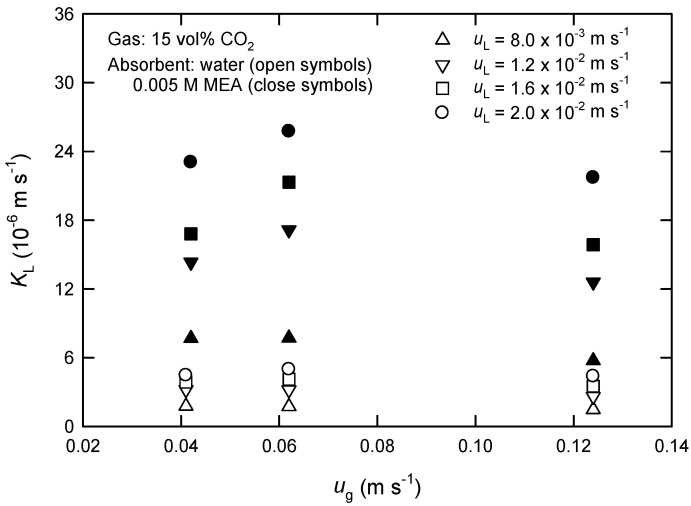
The overall mass-transfer coefficient at various gas-phase velocities using water and 0.005 M of MEA solution as absorbent liquids in extra-flow 2.5 × 8 module.

**Figure 4:**
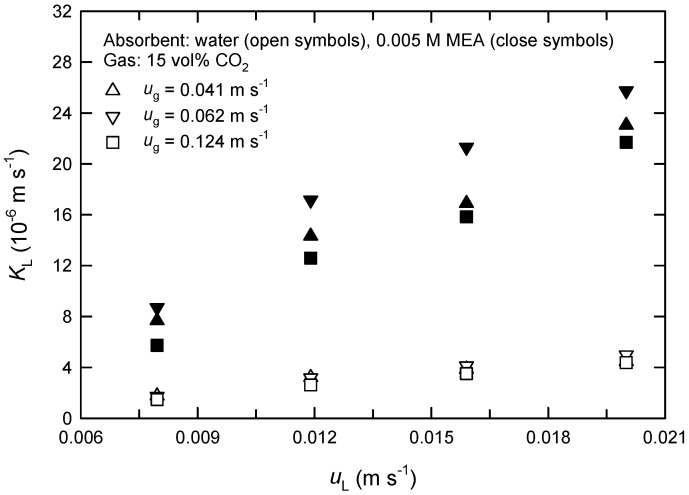
The overall mass-transfer coefficient at various liquid-phase velocities using water and 0.005 M of MEA solution as absorbent liquids in extra-flow 2.5 × 8 module.

The measured changes of CO_2_ concentrations in both phases between the inlet and outlet of the module were used for the calculation of the rates of CO_2_ absorption *R*_A_ and the overall mass-transfer coefficients *K*_L_ by Equation (1). Figure 3 and Figure 4 show the influences of fluid velocities on the *K*_L_ values in PP hollow fibers using water and 0.005 M of MEA solution as absorbent liquids. As expected, *K*_L_ increases initially with increasing gas-phase velocity *u*_g_ but then decreases (Figure 3). The decreased *K*_L_ is likely because the retention time of gas reduces when *u*_g_ is increased. It is noted that the flux of CO_2_ absorption, *J*_A_, at higher *u*_g_ is still larger than that at low *u*_g_. On the other hand, the *K*_L_ value always increases with increasing liquid-phase velocity *u*_L_ using both water and MEA solutions as absorbent liquids as shown in Figure 4. Furthermore, the influence of gas-phase flow rate in chemical absorption (i.e., MEA) is more significant than that in physical absorption (i.e., water).

Yeon et al. [15] have ever investigated the absorption of CO_2_ in PVDF and PTFE hollow fiber modules using single MEA solution. They also found that the flux of CO_2_ increases with an increase in liquid-phase velocity, and that initially increases with an increase in gas-phase velocity. However, it has been reported according to theoretical analysis that the flux of CO_2_ absorption by MDEA solution is virtually unaffected by liquid-phase velocity [16]. This is likely due to the variations of membrane configuration and the range of gas-phase velocity. Under the present conditions studied, the difference of *K*_L_ scales by 8 times between the systems using water and MEA solution is due to the characteristics of physical and chemical absorption.

It is expected that the experimental *K*_L_ values evaluated by Equation (1) should vary with MEA concentration in chemical absorption processes. This fact makes the proposed kinetic model, Equation (12), rather complicated and practically unpromising. For simplicity, accordingly, the value of *K*_L_ evaluated from the physical absorption process is applied throughout this work for model predictions, regardless of physical or chemical absorption processes.

### 4.2. Effect of Operation Parameters on CO_2_ Removal with Absorbent Recycling

**Figure 5:**
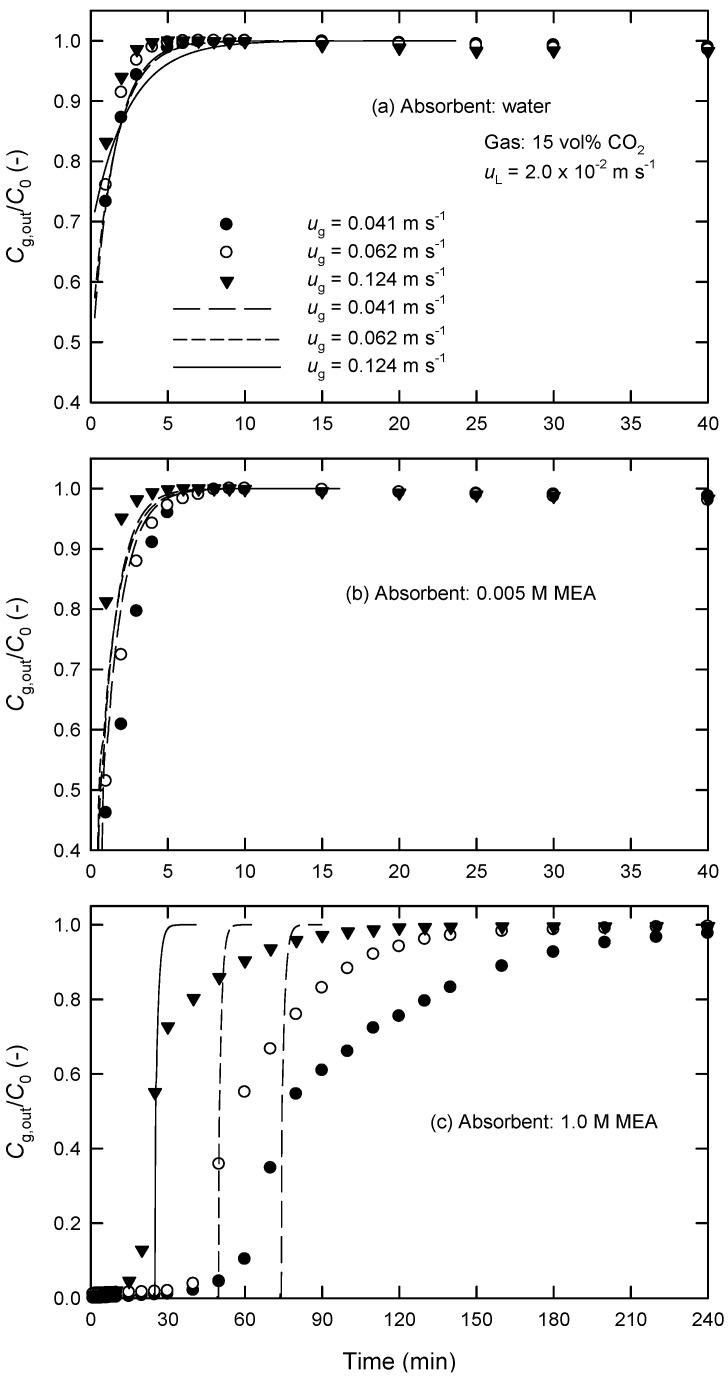
Effect of gas-phase velocity on the time changes of *C*_g,out_/*C*_0_ at *u*_L_ = 2.0 × 10^−2^ m s^−1^ using (**a**) water, (**b**) 0.005 M of MEA, and (**c**) 1.0 M of MEA as absorbent liquids in extra-flow 2.5 × 8 module (the solid and dashed curves are calculated by the proposed kinetic model).

**Figure 6:**
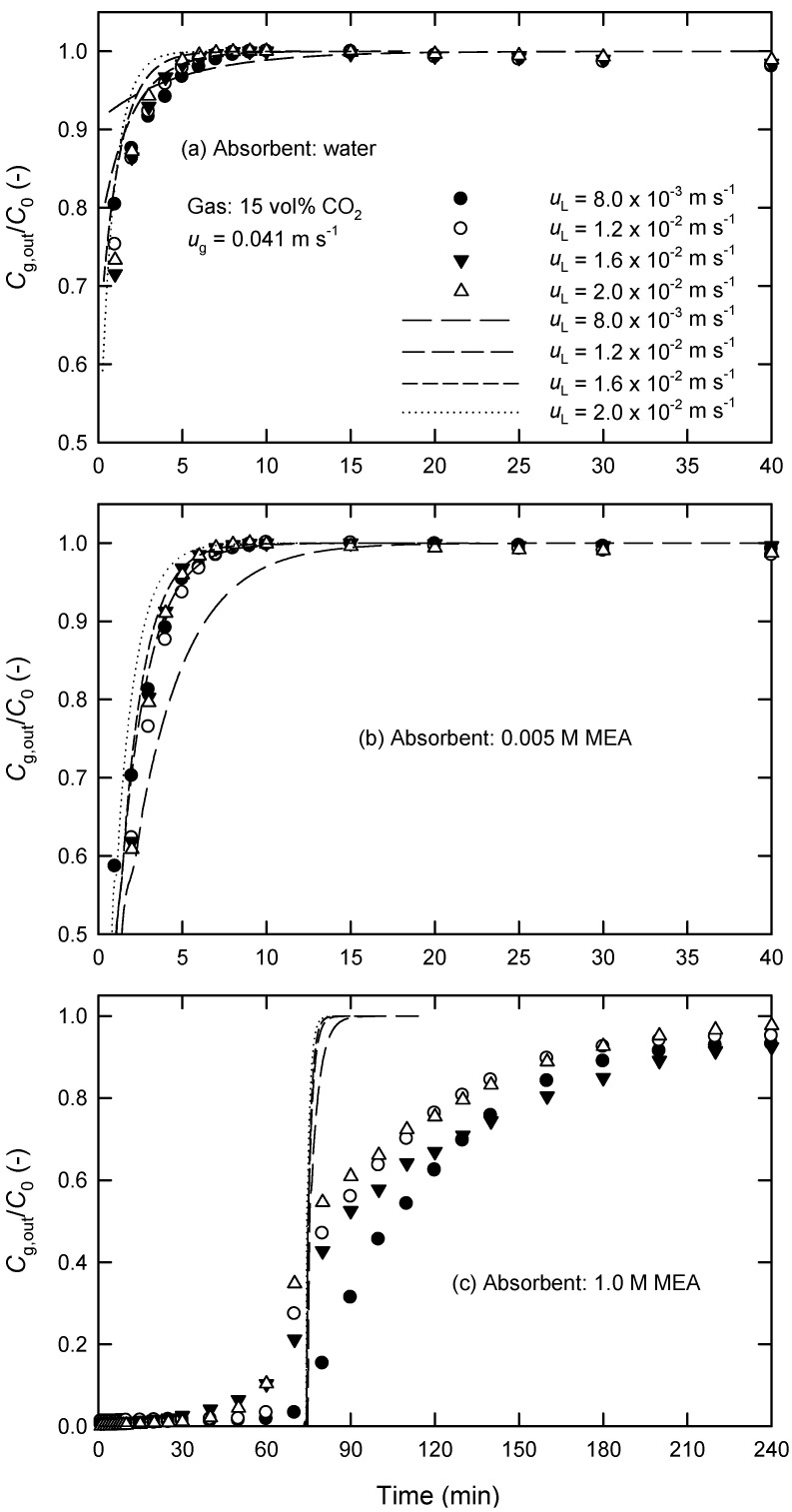
Effect of liquid-phase velocity on the time changes of *C*_g,out_/*C*_0_ at *u*_g_ = 4.1 × 10^−2^ m s^−1^ using (**a**) water, (**b**) 0.005 M of MEA, and (**c**) 1.0 M of MEA as absorbent liquids in extra-flow 2.5 × 8 module (the solid and dashed curves are calculated by the proposed kinetic model).

The time changes of the concentrations of CO_2_ in the outlet of the module under various gas- and liquid-phase velocities using different absorbent liquids are shown in Figure 5 and Figure 6. At a specific time (that is, a specific position along the axial direction in HFMCs), the steady state is assumed to be calculated *J*_A_ from Equations (3) and (4) in the case of using water as well as Equations (6) and (11) in the case of using MEA solution using the MATLAB program. Then, we can calculate the exit concentrations of CO_2_ in the water and MEA solution, *C*_L,out_ by Equations (5) and (12), respectively. The exit concentration of CO_2_ in the gas can obtained using Henry’s law, *P*_g,out_ = *C*_L,out_ × *H*, and ideal gas law. At the next time, similar procedures are repeated. Consequently, we can obtain the modeled results of *C*_g,out_ at different times.

It is found that CO_2_ can be removed by pure water only within 4 min, and the time elapsed when *C*_g,out_/*C*_0_ reaches unity becomes shorter with increasing *u*_g_. It is expected that the absorption of CO_2_ by recycling absorbent liquids is faster at a higher *u*_g_. This phenomenon is more obvious in the case of aqueous MEA solution (Figure 5b,c). The time required when *C*_g,out_ = *C*_0_ for MEA solution is much longer than that for water. This is because the CO_2_ loading of the MEA solution, particularly at 1 M of MEA, is larger than that of water.

However, the effect of liquid-phase velocity *u*_L_ on CO_2_ removal is not so apparent, as shown in Figure 6a,b. Figure 6c shows that the time required for *C*_g,out_ to reach *C*_0_ decreases with increasing *u*_L_ at high MEA concentrations, which is mainly a result of a larger mass-transfer coefficient at higher *u*_L_. The loading of CO_2_ of absorbent liquids is depleted more quickly when the rate of absorption increases. Furthermore, it is inferred that the resistance of liquid phase mass transfer is always important in chemical absorption, particularly at high MEA concentrations.

### 4.3. Validity of the Proposed Kinetic Model

Figure 5 and Figure 6 also show the calculated results (solid and dashed curves) in the present PP hollow-fiber module. It is found that the measured results agree reasonably well with the calculated ones using water and low MEA concentrations as shown in Figure 5a,b and Figure 6a,b. The standard deviation (SD) is less than 11% (mostly 7%), which is defined by

(13) $$\mathrm{SD}\left(\%\right)=100\times {\left\{\underset{N}{\sum }{\left[1-\left(C_{\mathrm{calc}}/C_{\mathrm{expt}}\right)\right]}^{2}/\left(N-1\right)\right\}}^{1/2}$$

where the subscripts ‘calc’ and ‘expt’ are the calculated and measured values, respectively, and *N* is the number of data points. The agreement is also acceptable using 0.05 M of MEA solution, where SD is less than 15%. At higher MEA concentrations (e.g., 1.0 M of MEA in Figure 5c and Figure 6c), the large SD (more than 100%) is likely attributed to the ignorance of membrane wetting effect in this model. However, the fact that the predicted lines still approximately pass through the “center of symmetry” of the measured curves, as shown in Figure 5c and Figure 6c, can be understood by a uniform distribution of pore wetting within the membrane. Although the large SD values found in Figure 5c and Figure 6c, this simple model would still reveal the time required for *C*_g,out_ to reach *C*_0._

**Figure 7:**
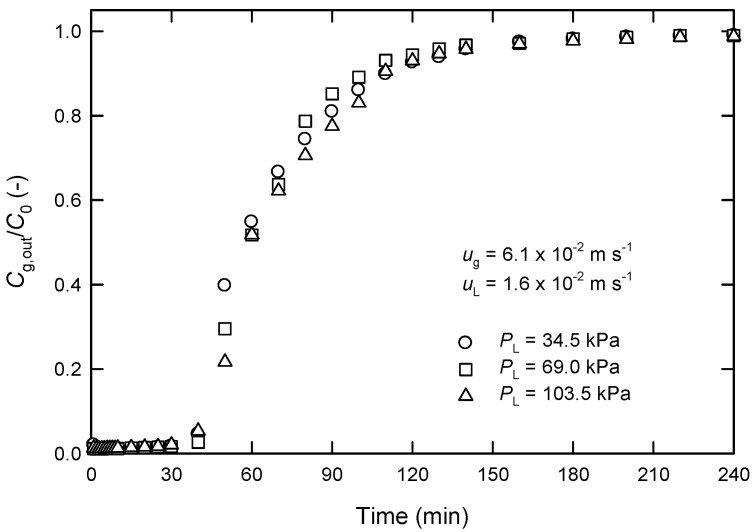
Effect of liquid-phase pressure in the shell of the module *P*_L_ on time changes of *C*_g,out_/*C*_0_ at a fixed gas-phase pressure of 20 kPa in extra-flow 2.5 × 8 module using 0.005 M of MEA as absorbent liquid.

It is recognized that the wetting ratio of the membrane is a crucial factor on the mass transfer resistance during the absorption of gases in HFMCs. In general, the wetting ratio is dominantly affected by the pressure difference between the gas- and liquid-phases and hydrophobicity of absorbent. Experiments with various liquid-phase pressures *P*_L_ at a fixed gas-phase pressure (20 kPa) were also conducted in this work to understand such a factor on the rate of CO_2_ absorption. It is found from Figure 7 that the effect of such pressure differences on CO_2_ removal can be neglected using 0.005 M of MEA solution as absorbent liquid under the conditions investigated.

It has been reported that neglecting axial diffusion will result in a much smaller CO_2_ concentration along the length of the fiber and, thus, a higher rate of absorption. This is the case at low Peclet numbers (=*u*_g_*L*/*D*_A,g_), where *D*_A,g_ is the diffusivity of CO_2_ in gas phase (= 1.51 × 10^−5^ m^2^ s^−1^) [25]; However, such an effect becomes less when the Peclet number increases (for example, >50) [26]. In the present system, the axial diffusion plays a negligible role in kinetic modeling because the Peclet number is larger than 500.

**Figure 8:**
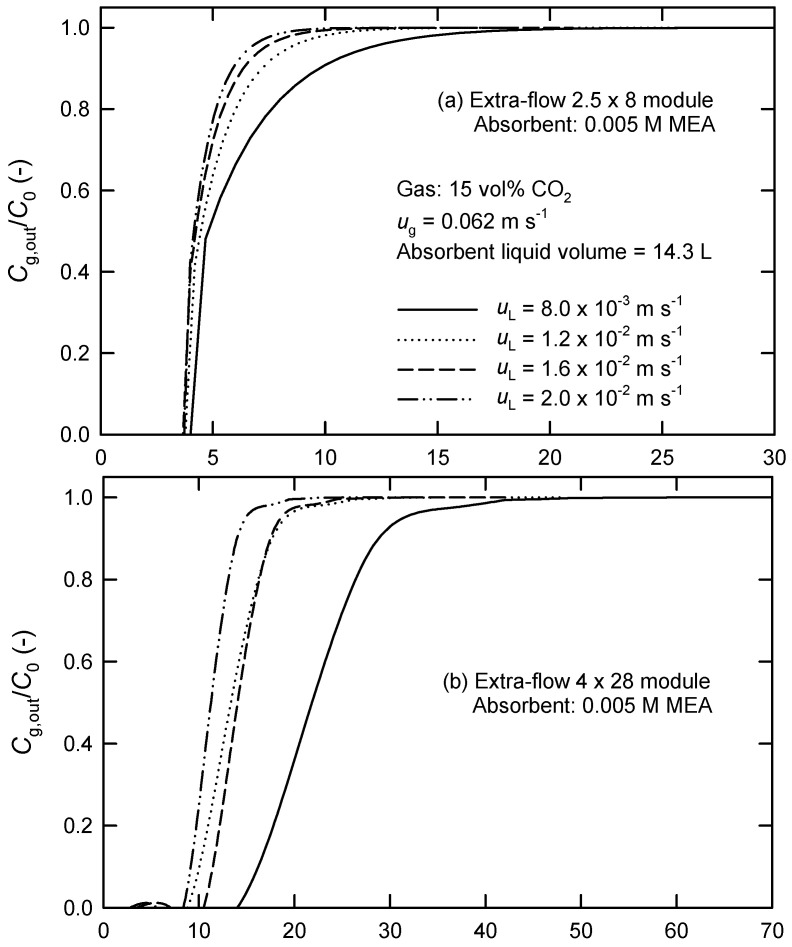

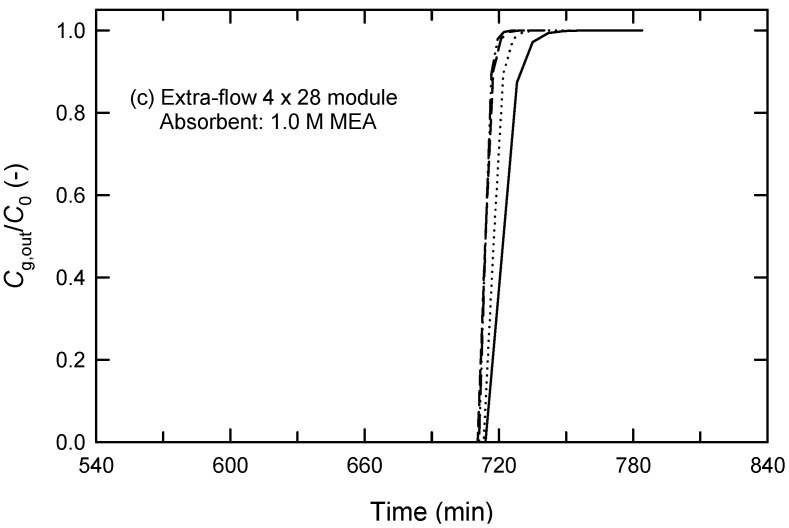
Effect of liquid-phase velocity on the time changes of *C*_g,out_/*C*_0_ at *u*_g_ = 0.062 m s^−1^ using (**a**) water, (**b**) 0.005 M of MEA, and (**c**) 1.0 M of MEA as absorbent liquids in extra-flow 2.5 × 8 and 4 × 28 modules.

Once the validity of the proposed simplified model was confirmed, an attempt was made to understand the role of the size of hollow fibers. Figure 8 shows the calculated results in a large HFMC, extra-flow 4 × 28 module (fiber length 620 mm, contact area 20 m^2^, fiber inner diameter 0.22 mm), whose characteristics are listed in Table 1. In contrast to the case of extra-flow 2.5 × 8 module (fiber length 190 mm, contact area 1.4 m^2^, fiber inner diameter 0.22 mm), the effect of *u*_L_ on the time required for *C*_g,out_ reaching *C*_0_ in a larger module is more significant (Figure 8a,b). It is noted that the volume of absorbent liquid was kept the same (14.3 L) in both modules. Moreover, the use of larger amount of absorbent liquid leads to a higher efficiency of CO_2_ absorption, as compared in Figure 6b vs. Figure 8b and Figure 6c vs. Figure 8c.

Given the absence of experimental data using a larger extra-flow 4 × 28 module, this note is necessarily highly prospective. Additionally, smaller modules were used in the literature and in this work; they could deliver good agreement between the measured and predicted results.

### 4.4. Determination of Effective Fiber Length

**Figure 9:**
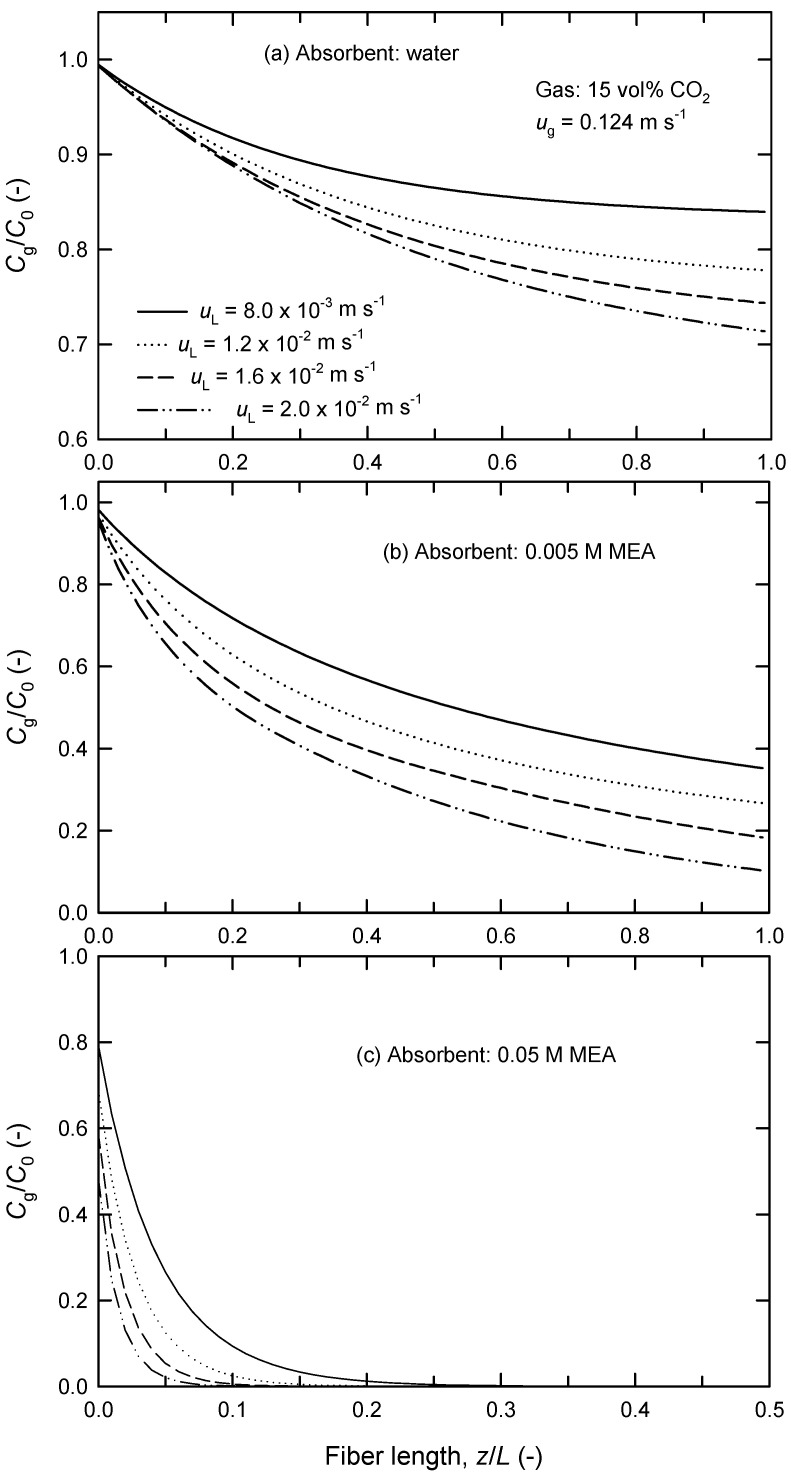
Effect of liquid-phase velocity on the variation of CO_2_ removal along the length of the fiber at *u*_g_ = 0.124 m s^−1^ using (**a**) water, (**b**) 0.005 M of MEA, and (**c**) 0.05 M of MEA as absorbent liquids in extra-flow 2.5 × 8 module.

On the other hand, the present model enables us to predict the concentration change of CO_2_ along the fiber from the gas inlet to the outlet. It is found from Figure 9a,b that the CO_2_ concentration in the outlet of the module, *C*_g,out_, is higher when liquid-phase velocity *u*_L_ is smaller. In addition, *C*_g,out_ becomes zero for all *u*_L_ values tested when *u*_g_ = 0.124 m s^−1^ using 0.05 M MEA solution as an absorbent liquid (Figure 9c). For example, the dimensionless length of the fiber (*z*/*L*) needed for nearly complete removal of 15% CO_2_ is 0.2 (whereas *z*/*L* = 0.06 when *C*_g_/*C*_0_ = 0.01) using 0.05 M of MEA solution as *u*_g_ = 0.124 m s^−1^ and *u*_L_ = 2.0 × 10^−2^ m s^−1^, as shown in Figure 9c. We call this the “effective” fiber length, *L*_eff_. It is also found that *L*_eff_ remarkably reduces when more concentrated MEA solution is used as an absorbent liquid. Zhang et al. [27] have actually reported that CO_2_ absorption by aqueous DEA solution in HFMC (Celgard MiniModule^®^ 0.75 × 5 module, X-50 type fibers; fiber length 113 mm, contact area 0.09 m^2^) is mainly conducted in the front segments near the inlet. For example, 20 vol% CO_2_ in CO_2_-N_2_ mixture (*u*_g_ = 0.032 m s^−1^) is mainly absorbed by 2 M of DEA (*u*_L_ = 0.15 m s^−1^) in the segments up till *z*/*L* = 0.6 while CO_2_ absorption is negligible in the rest of the segments. Although increasing *u*_g_ in the range 0.032–0.090 m s^−1^ can increase *L*_eff_, approximately 20% of the total length has little absorption capacity. They thus concluded that increasing the length of the module is not an effective way to enhance CO_2_ absorption when the module is longer than *L*_eff_.

The calculated results of *L*_eff_ in extra-flow 2.5 × 8 module under different conditions are listed in Table 2. It is evident that *L*_eff_ decreases with increasing liquid-phase velocity *u*_L_ and absorbent concentration but increases with increasing gas-phase velocity *u*_g_. Similarly, Table 3 shows the results of *L*_eff_ in extra-flow 4 × 28 module.

Evidently, such a simplified model can predict a suitable membrane length of the fiber for desired CO_2_ removal or estimate an appropriate absorbent concentration for fixed length of the fiber. Generally speaking, the knowledge of effective fiber length is important for economical applications of HFMC processes. The relationship among effective fiber length, gas-phase flow rate, and absorbent concentration for fixed emissive CO_2_ concentration is crucial for the HFMC process to scale up.

## 5. Conclusions

The absorption of CO_2_ from CO_2_-N_2_ mixtures using water and monoethanolamine (MEA) solution as absorbent liquids in hollow-fiber polypropylene membrane contactors has been experimentally and theoretically investigated. Through the use of the overall mass-transfer coefficients purely evaluated from physical absorption (i.e., water), a simplified model that considers mass balance equations and membrane non-wetting acceptably followed chemical absorption process (i.e., MEA); this is particularly true for a concentration of MEA lower than 0.05 M, where the standard deviation was less than 15%. The time changes of the gas-phase CO_2_ concentration in the outlet of the module, as well as the gas-phase CO_2_ concentration along the fiber within the module, could be obtained by iterative calculation. Model prediction of the effective length of the fiber, defined as the length from the inlet where the gas-phase CO_2_ concentration reduced to zero, depended not only on the contact area of the module but also on the total length of the fiber. Although membrane wetting was more serious at higher MEA concentrations (e.g., 1.0 M), on average, model predictions still adequately described the dynamics of CO_2_ absorption process in hollow-fiber membrane contactors.

## Figures and Tables

**Table 1:** Characteristics of HFMCs used in this work (Hoechst Celanese Liqui-Cel extra-flow model with central baffle).

Description	Extra-Flow 2.5 × 8 Module	Extra-Flow 4 × 28 Module
Shell material	Polypropylene	Polypropylene
Shell length (mm)	203	704
Shell outer diameter (mm)	77	127
Shell inner diameter (mm)	63	111
Shell hydraulic diameter (mm)	4.7	--
Fiber material	Celgard X-50 polypropylene	Celgard X-50 polypropylene
Number of fibers	~10,200	--
Fiber length, *L* (mm)	190	620
Fiber inner diameter (μm)	220	220
Fiber outer diameter (μm)	300	300
Fiber membrane surface area, *A*_T_ (m^2^)	1.4	20
Fiber membrane pore size (μm)	0.04	0.04
Fiber membrane porosity	0.4	0.4
Effective area/volume (cm^2^ cm^−3^)	25.5	36.4

**Table 2:** Effective fiber length (in mm) for CO_2_ absorption by aqueous MEA solutions in extra-flow 2.5 × 8 module (*L* = 190 mm).

*u*_L_ (m s^−1^)	*u*_g_ = 0.041 m s^−1^			*u*_g_ = 0.062 m s^−1^			*u*_g_ = 0.124 m s^−1^		
	0.005 M	0.01 M	0.1 M	0.005 M	0.01 M	0.1 M	0.005 M	0.01 M	0.1 M
7.96 × 10^−3^	192.3	189.3	5.0	232.0	228.2	8.3	257.4	252.0	29.4
1.10 × 10^−2^	153.9	146.9	2.6	196.3	193.4	4.0	241.8	238.2	12.0
1.59 × 10^−2^	146.1	135.8	2.1	184.0	180.5	3.0	229.4	225.6	8.2
2.00 × 10^−2^	141.9	126.6	1.8	177.8	174.9	2.4	225.0	221.9	6.1

**Table 3:** Effective fiber length (in mm) for CO_2_ absorption by aqueous MEA solutions in extra-flow 4 × 28 module (*L* = 620 mm).

*u*_L_ (m s^−1^)	*u*_g_ = 0.041 m s^−1^		*u*_g_ = 0.062 m s^−1^		*u*_g_ = 0.124 m s^−1^	
	0.005 M	0.1 M	0.005 M	0.1 M	0.005 M	0.1 M
7.96 × 10^−3^	54.6	1.3	58.4	6.2	64.7	9.1
1.10 × 10^−2^	43.7	0.7	48.8	1.2	61.0	3.1
1.59 × 10^−2^	41.8	0.6	45.2	1.2	57.7	2.1
2.00 × 10^−2^	41.0	0.5	43.5	1.2	56.6	1.6

## Data Availability

The data presented in this study are available on request from the corresponding author.

## List of Symbols

*A*_T_ = effective contact area (m^2^)
*C =* concentration of components (mol m^−3^)
*C*_0_ = initial CO_2_ concentration in gas phase (mol m^−3^)
*H* = Henry’s law constant of CO_2_ (m^3^ Pa mol^−1^)
*J*_A_ = flux of CO_2_ (mol m^−2^ s^−1^)
*K*_L_ = overall mass-transfer coefficient based on liquid phase (m s^−1^)
*k*_-1_ = first-order rate constant for the reverse reaction defined in Equation (8) (m^3^ mol^−1^ s^−1^)
*k*_2_ = second-order rate constant for the forward reaction defined in Equation (8) (m^3^ mol^−1^ s^−1^)
*k*_2,MEA_ = second-order rate constant defined in Equation (8) (m^3^ mol^−1^ s^−1^)
*k*_B_ = second-order rate constant for base B defined in Equation (9) (m^3^ mol^−1^ s^−1^)
*k*_w_ = *k*_B_ when B is water (m^3^ mol^−1^ s^−1^)
*k*_MEA_ = *k*_B_ when B is MEA (m^3^ mol^−1^ s^−1^)
*L* = fiber length (mm)
*L*_eff_ = effective fiber length for CO_2_ absorption (mm)
*N*_z_ *=* the number of grids in the computational domain in the axial direction
*Q*_L_ = volumetric flow rate of liquid phase (m^3^ s^−1^)
*r*_A_ = rate of reaction between CO_2_ and MEA defined in Equation (10) (mol m^−3^ s^−1^)
*R*_A_ = overall rate of CO_2_ absorption (mol s^−1^)
*R* = gas constant (=8.314 J mol^−1^ K^−1^)
SD = standard deviation defined in Equation (13) (%)
*u*_g_ = linear flow velocity of gas phase (m s^−1^)
*u*_L_ = linear flow velocity of liquid phase (m s^−1^)
*V*_shell_ = volume of shell side (m^−3^)
*z* = axial direction of the fiber (m)
Subscripts
A = CO_2_ gas
B = base (amine, H_2_O, etc.)
g = CO_2_ in gas phase
in = inlet
L = CO_2_ in liquid phase
lm = logarithm mean
out = outlet
w = water
